# Protein modeling to assess the pathogenicity of rare variants of *SERPINA1* in patients suspected of having Alpha 1 Antitrypsin Deficiency

**DOI:** 10.1186/s12881-019-0852-5

**Published:** 2019-07-15

**Authors:** Friedrich Kueppers, Mark D. Andrake, Qifang Xu, Roland L. Dunbrack, Joannah Kim, Christopher L. Sanders

**Affiliations:** 1 Department of Thoracic Medicine and Surgery, Lewis Katz School of Medicine, Temple University, Philadelphia, PA USA; 2 Molecular Therapeutics Program, Institute for Cancer Research, Fox Chase Cancer Center, Philadelphia, Pennsylvania USA; 3 Biocerna LLC, Fulton, Maryland USA

**Keywords:** Alpha 1 Antitrypsin Deficiency, *SERPINA1*, Rare variants, Protein modeling

## Abstract

**Background:**

Alpha 1 Antitrypsin (AAT) is a key serum proteinase inhibitor encoded by *SERPINA1*. Sequence variants of the gene can cause Alpha 1 Antitrypsin Deficiency (AATD), a condition associated with lung and liver disease. The majority of AATD cases are caused by the ‘Z’ and ‘S’ variants – single-nucleotide variations (SNVs) that result in amino acid substitutions of E342K and E264V. However, *SERPINA1* is highly polymorphic, with numerous potentially clinically relevant variants reported. Novel variants continue to be discovered, and without reports of pathogenicity, it can be difficult for clinicians to determine the best course of treatment.

**Methods:**

We assessed the utility of next-generation sequencing (NGS) and predictive computational analysis to guide the diagnosis of patients suspected of having AATD. Blood samples on serum separator cards were submitted to the DNA_1_ Advanced Screening Program (Biocerna LLC, Fulton, Maryland, USA) by physicians whose patients were suspected of having AATD. Laboratory analyses included quantification of serum AAT levels, qualitative analysis by isoelectric focusing, and targeted genotyping and NGS of the *SERPINA1* gene. Molecular modeling software UCSF Chimera (University College of San Francisco, CA) was used to visualize the positions of amino acid changes as a result of rare/novel SNVs. Predictive software was used to assess the potential pathogenicity of these variants; methods included a support vector machine (SVM) program, PolyPhen-2 (Harvard University, Cambridge, MA), and FoldX (Centre for Genomic Regulation, Barcelona, Spain).

**Results:**

Samples from 23 patients were analyzed; 21 rare/novel sequence variants were identified by NGS, including splice variants (*n* = 2), base pair deletions (*n* = 1), stop codon insertions (n = 2), and SNVs (*n* = 16). Computational modeling of protein structures caused by the novel SNVs showed that 8 were probably deleterious, and two were possibly deleterious. For the majority of probably/possibly deleterious SNVs (I50N, P289S, M385T, M221T, D341V, V210E, P369H, V333M and A142D), the mechanism is probably via disruption of the packed hydrophobic core of AAT. Several deleterious variants occurred in combination with more common deficiency alleles, resulting in very low AAT levels.

**Conclusions:**

NGS and computational modeling are useful tools that can facilitate earlier, more precise diagnosis, and consideration for AAT therapy in AATD.

**Electronic supplementary material:**

The online version of this article (10.1186/s12881-019-0852-5) contains supplementary material, which is available to authorized users.

## Background

Alpha 1 Antitrypsin (AAT) is a glycoprotein normally present in human blood at a concentration between 90 and 180 mg/dL [1]. It is encoded by the *SERPINA1* gene that is located on the long arm of chromosome 14 (cytogenetic location: 14q32.13); the gene encompasses 12.2 kb, containing 4 exons and 3 introns [2]. AAT is an effective inhibitor of serine proteinases, in particular leukocytic elastase; in this capacity it exerts a protective function on various tissues, especially the lungs, against proteolytic/elastolytic damage [3].

AAT is a highly polymorphic protein; over 70 sequence variants have been reported to be clinically significant and over 500 single-nucleotide variations (SNVs) identified in mutation databases. Some variants are common in certain populations such that their frequency may be maintained by a heterozygous selective advantage [4]. Common alleles that fit this definition, including PI*Z and PI*S, are frequent in Northern Europe and Spain/Portugal respectively [5].

The nomenclature (Z, S, M etc.) refers to a lettering system in which the normal common allele is designated PI*M, and other letters refer to the isoelectric point of the protein in a pH gradient established by isoelectric focusing (IEF) – a common method used to identify AAT variants [1]. Certain relatively common variants, in particular PI*Z and S, are associated with low levels of AAT in the circulation [1]. The Z and S alleles are caused by E342K and E264V substitutions, respectively; both cause misfolding and polymerization (to a lesser extent with the S allele) of AAT [2]. The Z mutation also results in retention of polymerized AAT in hepatocytes, leading to severe deficiency and liver disease, and is of special clinical interest.

Among patients of European ancestry with chronic obstructive pulmonary disease (COPD; including emphysema), 1–3% have been found to have Alpha 1 Antitrypsin Deficiency (AATD), usually due to homozygosity for PI*Z [6]. There are, however, less common deficiency alleles that can also be associated with reduced AAT levels and lung disease [7–9]. As testing and screening become more widely used, more variants associated with low AAT levels continue to be uncovered [10]. Primarily, the improved identification of rare/novel variants is due to the increased use of DNA sequencing. In particular, next-generation sequencing (NGS), a far higher-throughput technology than Sanger sequencing [11], has the potential to improve the diagnosis of AATD through the enhanced detection of rare/novel variants [12].

We report a number of rare/novel *SERPINA1* sequence variants detected with the use of NGS in a US-wide AATD targeted detection program. To characterize the potential deleterious effects of these variants, we utilized a number of molecular modeling analyses. Our aim was to cover the whole spectrum from the nucleotide base change to the altered protein structure, and predict the clinical consequences to the patient.

## Methods

### Subjects

Patients were recruited from the Lewis Katz School of Medicine, Temple University, Philadelphia, Pennsylvania (*n* = 4) or through nationwide physician referral to the DNA_1_ Advanced Alpha-1 Screening™ program (developed and performed by Biocerna LLC, Fulton, Maryland, USA, on behalf of CSL Behring, King of Prussia, Pennsylvania, USA; *n* = 19). Blood samples on serum separator cards were collected through routine clinical testing by the treating physician and sent to Biocerna for AATD screening.

Patients were included in this study if discordance existed between the patient’s AAT level and the targeted genotyping results. For these patients, NGS was used to identify rare or potentially novel genetic variants. Consent for use of laboratory data for research purposes was provided by all patients included in this study. The study was approved by the Institutional Review Board of Temple University, Philadelphia, PA.

### Laboratory analyses

Data on antigenic serum AAT and c-reactive protein levels, AAT phenotype by IEF, and genetic analyses by targeted real-time polymerase chain reaction and NGS were collected for patients included in this study.

Serum AAT levels were assessed in all patients. For the four patients referred from Lewis Katz School of Medicine, quantitative analysis of antigenic serum AAT was performed by radial immunodiffusion (normal range: 150–400 mg/dL) at Temple University. For the remaining patients, antigenic AAT and CRP levels were assessed using immunoturbidimetry (normal range: 90–200 mg/dL and < 5 mg/L, respectively) [Roche™ AAT2 and C-Reactive Protein gen 3 immunoassay; Basel, Switzerland] at Biocerna.

All genetic and IEF analyses were performed centrally at Biocerna. Initial qualitative assessment of AATD genotype was by real-time polymerase chain reaction targeted genotyping (TaqMan**®**: Thermo Fisher Scientific, Waltham, MA). Phenotype was investigated using IEF (Hydragel 18 A1AT IEF isofocusing kit, Sebia USA, Norcross, GA).

NGS methodology includes sequencing of *SERPINA1* 5′ and 3′ untranslated regions (UTRs), the promotor region, coding exons, introns, and splice sites. Specific target regions of the *SERPINA1* gene were amplified using the Ion AmpliSeq™ Custom Primer Pools (Thermo Fisher Scientific). The primer pools included a total of 52 amplicons containing unique PCR primers to amplify specific target sequences in each template DNA. Library preparation was performed using Ion AmpliSeq™ Library Kit 2.0–96 LV (Thermo Fisher Scientific). Sample identity was maintained using unique Ion Xpress™ Barcode Adapters (Thermo Fisher Scientific), which allowed for multiplexed sequencing analysis. Emulsion PCR was used to clonally amplify the library DNA onto the Ion Sphere™ Particles (ISP). Following ISP template amplification, the ISP-enriched template-positive library was loaded onto an Ion 314™ Chip Kit v2 (Thermo Fisher Scientific). The Ion PGM instrument was used to sequence the combined library.

### Computational modeling and variant predictions

**Fig. 1:**
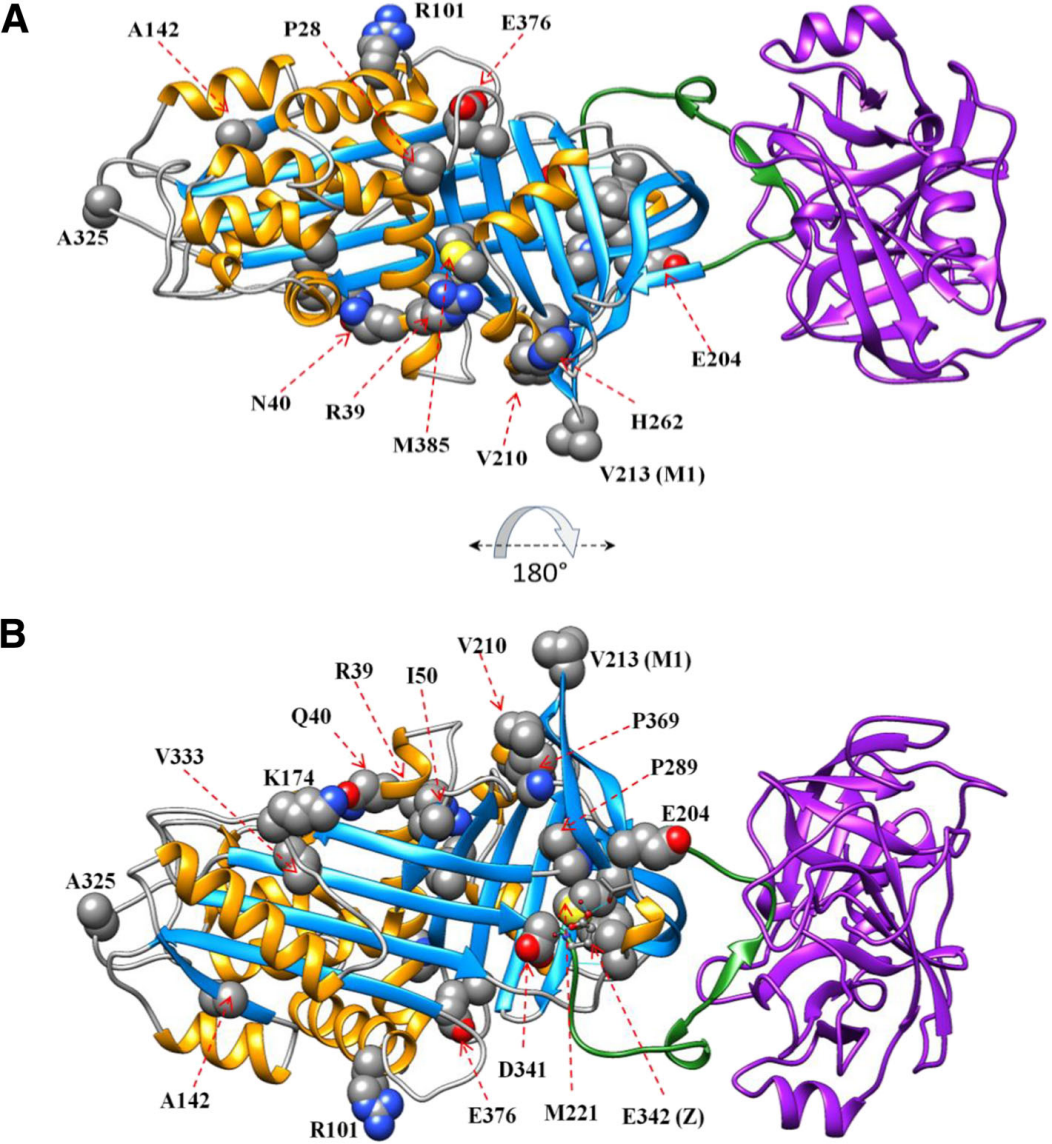
Structure of AAT indicating the location of missense residues. The AAT protein (PDB code 1OPH) is shown in ribbon representation coloring according to secondary structural elements (alpha helices shown in orange, beta strands shown in light blue), and the position of missense changes showing the wildtype residue in sphere representation and labeled with the residue name and position. The purple ribbon protein is trypsinogen. The stretch of amino acids that comprise the reactive center loop are shown in green ribbon representation. A = front view; B = rear view (rotated 180 degrees about the x-axis). AAT, Alpha 1 Antitrypsin

**Fig. 2:**
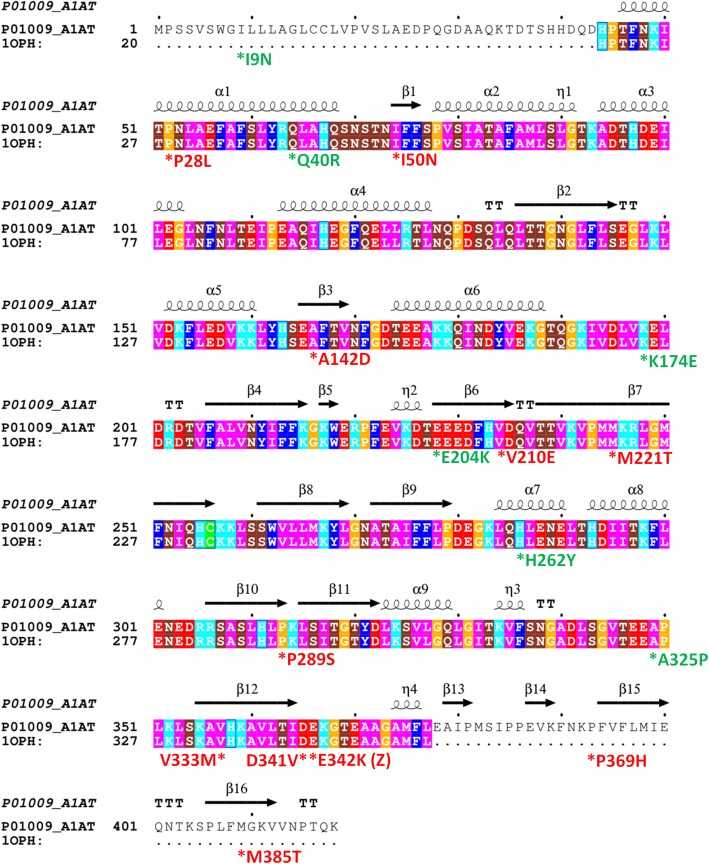
Primary and secondary structure of AAT. While the top row sequence represents Uniprot numbering including the signal peptide, the bottom sequence row uses the conventional numbering for AAT found in the broad literature, and used throughout this manuscript. Color coding of residues are according to chemical properties, and display of secondary structural elements (arrow for β-strand, curl for α-helix as extracted from PDB code 1OPH) are shown above the sequences using the software package ESPript [13]. The positions of the novel alleles reported in this manuscript are marked by an asterisk below the residue, and include the variant residue found. Those that are probably or possibly deleterious are colored red, and those that are possibly or probably neutral are colored green. Numbering of the variants, apart from I9N, does not include the 24 amino acid precursor. AAT, Alpha 1 Antitrypsin

To visualize and map the locations of sequence variants in the AAT proteins, molecular modeling software UCSF Chimera (University College of San Francisco, CA) was used (Fig. 1). A linear diagram of the AAT amino acid sequence with mutation locations was also prepared (Fig. 2) using the software package ESPript [13].

#### Predicting pathogenicity

For all missense SNVs, NGS sequencing information was inputted into a support vector machine (SVM) model. This model combines multiple features, including both sequence- and structure-based information, to calculate the probability that a given missense change is pathogenic [14]. The SVM score is in the range of zero to 1.0, with a threshold for a deleterious change set at a value of 0.5 and above. Further details concerning the SVM model utilized are outlined in the Additional file 1.

In addition to the SVM predictions, two other computational predictors of pathogenicity were utilized. First, Gibbs free energy changes (ΔΔG) associated with amino acid substitutions were calculated using the PositionScan function of the FoldX suite [Centre for Genomic Regulation, Barcelona, Spain] [15]. ΔΔG is the difference in free energy (in kcal/mol) between a mutant and wildtype protein. A mutation with ΔΔG > 0 will destabilize the structure, while a mutation with negative ΔΔG stabilizes the structure. A common threshold used to indicate that a mutation has a significant destabilizing effect is ΔΔG > 1 kcal/mol [16], and was therefore set as the threshold for pathogenicity in the present report. Second, PolyPhen-2 program (http://genetics.bwh.harvard.edu/pph2/index.shtml; version 2.2.2, Harvard University, Cambridge, MA) was also used to predict the pathogenicity of all missense SNVs. PolyPhen-2 uses an iterative greedy algorithm, informed by exposure to known damaging and non-damaging SNVs, and calculates the Naïve Bayes posterior probability that a given mutation is damaging [17]. The Polyphen-2 score, also with a range of zero to 1.0 (but often stated as a percentage), has a qualitative ternary classification. Scores of 0.85, 0.85–0.15, and 0.15 are typically coded as “probably damaging”, “possibly damaging”, and “benign”, respectively.

Scores for all three predictive methods (SVM, FoldX, and PolyPhen-2) were grouped into the following classifications: probably deleterious (all three predictions as deleterious), possibly deleterious (two of the three predictions as deleterious), possibly neutral (only one of the three predictions as deleterious), or probably neutral (none of the three predictions as deleterious).

#### Benchmarking analysis of SVM predictions

We confirmed the effectiveness of the SVM method by performing benchmarking analysis against two datasets of known human *SERPINA1* pathogenic and benign variants sourced from ClinVar [18], and a third dataset composed of primate neutral variants (owing to the low number of benign human variants identified [*N* = 6]). To build the dataset of primate neutral variants, we ran PSI-BLAST with the Alpha 1 Antitrypsin (*SERPINA1*, A1AT_HUMAN) sequence as a query against a database of primate sequences from Uniprot (http://www.uniprot.org/). For each alignment, we identified all sequence differences between the human and primate sequence and filtered out sequence variants that were not surrounded by 2 conserved residues on either side (human and primate identical) and those adjacent to gaps within 3 residues. For each mutant, we used the search result with the highest sequence identity for that variant. In this manner we chose sequence variants that exist in the closest homologues first. We also checked the contacts of the human residue for each mutation in *SERPINA1* structure PDB: 3NE4 and filtered out sequence variants with one or more different contact residues. A contact is defined as a residue with at least one atomic distance less than 5 Å. This resulted in 35 neutral sequence variants garnered from primates with greater than 90% sequence identity.

#### Measurements of binary prediction of SERPINA1 variants

To further compare the accuracy of SVM predictions vs. PolyPhen-2, a number of statistical parameters were calculated. From the benchmarking data, we are able to obtain the number of true positives (TP), false positives (FP), true negatives (TN), and false negatives (FN). From these, we calculated the true positive rate (TPR), true negative rate (TNR), positive predictive value (PPV), and negative predictive value (NPV).

*True positive rate* (TPR)*, or sensitivity*: The proportion of experimental positives that are correctly predicted.

$$ \mathrm{TPR}=\frac{\mathrm{TP}}{\mathrm{TP}+\mathrm{FN}} $$

*True negative rate* (TNR)*, or specificity*: The proportion of experimental negatives that are predicted correctly.

$$ \mathrm{TNR}=\frac{\mathrm{TN}}{\mathrm{TN}+\mathrm{FP}} $$

*Positive predictive value* (PPV): The proportion of predicted positives that are predicted correctly.

$$ \mathrm{PPV}=\frac{\mathrm{TP}}{\mathrm{TP}+\mathrm{FP}} $$

*Negative predictive value* (NPV): The proportion of predicted negatives that are predicted correctly.

$$ \mathrm{NPV}=\frac{\mathrm{TN}}{\mathrm{TN}+\mathrm{FN}} $$

*Total accuracy* (ACC): The proportion of true results (both true positives and true negatives) among the total number of experimental cases.

$$ \mathrm{ACC}=\frac{\left(\mathrm{TP}+\mathrm{TN}\right)}{\left(\mathrm{P}+\mathrm{N}\right)}=\frac{\left(\mathrm{TP}+\mathrm{TN}\right)}{\left(\mathrm{TP}+\mathrm{FN}\right)+\left(\mathrm{FP}+\mathrm{TN}\right)} $$

*Balanced accuracy* (BACC): This is the average of true positive rate (TPR) and true negative rate (TNR).

$$ \mathrm{BACC}=\frac{1}{2}\left(\mathrm{TPR}+\mathrm{TNR}\right) $$

## Results

### Patient characteristics

Patient characteristics (*n* = 23), including AATD phenotype, AAT level, and sequencing findings, are displayed in Table 1. The male:female ratio was approximately 1:1, the age range was 34–87 years, and AAT levels ranged from 2 mg/dL to 160 mg/dL.

**Table 1:** Overview of patient characteristics and rare/novel sequence variants identified by next-generation sequencing

Patient ID	AAT serum level (mg/dL)	CRP (mg/dL)	Previous phenotypes	Age (yrs)	Sex	NGS DNA analysis		
						Novel mutant nucleotide changes	Consequence	Genotype
Splice variants								
2250	70	5.9	–	65	F	Novel splice variant: G > C at position +1 of intron 1C		E376D – M3 allele
24023	62.8	–		71	M	917 + 1G > A	Affects the normal mRNA splicing.	E376D – M3 allele
Deletions								
10724	52	7.3	M3	49	M	A347fs: Novel 1 bp deletion (1112delC) at position 347	Frameshift that extends the protein by 5 amino acids	E376D – M3 allele
Stop codons								
6326^e^	2	0	Z/M1	57	F	Q156X: C > T at Chr14:94849037 (GRCh.37.p13) c.538C > T	Insertion of stop codon at position 156	Q156X E342K – Z allele V213A – M1 allele
6376^e^	98	7	I	54	M	Q156X: C > T at Chr14:94849037 (GRCh.37.p13) c.538C > T	Insertion of stop codon at position 156	Q156X R39C – I allele
19771	91.4	–	M	57		G192Fs: 1 bp deletion at Chr14:94847477 (GRCh37.p13) c.647_647delG	Predicted to produce a premature stop codon at amino acid 214, leading to a premature termination on exon III	
Single-nucleotide variations								
CA97	112	–	M3 M2/4	65	F	GAG > AAG		E204K – rare^a^ E376D – M3 allele R101H – M2/M4 allele
1144	72	–	M1	61	M	CCC > TCC		P289S – rare^a^ V213A – M1 allele
2343	86	3.6	M1	60	F	ATC > AAC		I9N [includes precursor] – rare^a^ V213A – M1 allele
4293^d^	66	0.5	M1	54	M	CCC > CTC	Q_0Bellingham_ – insertion of stop codon at position 156	P28L – rare^a^ K217X – Q0_Bellingham_ V213A – M1 allele
5564^d^	67	1.6	M1	51	F	CCC > CTC	Q_0Bellingham_ – insertion of stop codon at position 156	P28L – rare^a^ K217X – Q0_Bellingham_ V213A – M1 allele
4668	78	2.2	M3	62	M	ATC > AAC		I50N (Pi_Tijarafe_) – rare E376D – M3 allele
9533	73	0.9	–	60	M	ATG > ACG		M385 T – rare^c^ M allele
10889	–	12.5	M3	34	F	CAG > CGG		Q40R – rare^a^ E376D – M3 allele
12642	89	0.6	M3	66	F	GAC > CTC		D341V – rare^b^ E376D – M3 allele P = L118 – no amino acid change
14271	47	0.6	Z/M1	61	F	ATG > ACG		M221 T – rare^a^ V213A – M1 allele E342K – Z allele
15230	34	1	Z/M1	72	M	GTG > GAG		V210E (N_cambodia_/Pierre-bénite) – rare^a^ V213A – M1 allele E342K – Z allele
17,657	160	1.5	M3/M4	87	M	AAG > GAG		K174E – NOVEL^a^ E376D – M3 allele R101H – M2/M4 allele
21034	121.2	–	–	47	F	CCC > CAC		P369H – rare E264V – S allele
21636	88.4	–	–	58	M	GTG > ATG		V333 M – rare^c^ E376D – M3 allele R101H – M4 allele
23523	118.6	–	–	48	F	GCA > CCA		A325P (Nvestenanova) – rare^a^ R223C – F allele E376D – M3 allele R101H – M4 allele
24319	79.3	–	–	57	F	GCC > GAC		A142D – rare^c^ E264V – S allele V213A – M1 allele
76430	74.8	–	–	59	M	CAC > TAC		H262Y – rare^c^ M allele

All SNVs are reported without the 24 amino acid precursor unless otherwise stated

^a^SNVs have been previously reported in dbSNP (E204K – rs199422208; P289S – rs779938258; Q40R – rs763483402; M221 T – rs766260108; K174E – rs766034720; I9N [includes precursor] – rs1296175763; P28L – rs944607375)

^b^SNVs have been previously reported in dbSNP and linked to AATD (D314V – rs864622046)

^c^SNV previously reported in the literature (20–26) (H262Y – rs149537225; V333 M – 373,630,097; A142D – rs142942004; V210E – rs746197812; M385 T – rs1488213352; A325P – rs376024688; I50N – rs1275309068)

^d^Familial samples from brother (4293) and sister (5564)

^e^Familial samples from brother (6376) and sister (6326)

*bp* Base pair, *dbSNP* Database of single nucleotide polymorphisms, *SNV* Single nucleotide variation

Clinical information on disease presentation was available from the four patients referred via Lewis Katz School of Medicine: patient CA97 presented with a cerebral aneurysm, patient 1144 presented with evidence of emphysema, and patient 4668 presented with chronic inflammatory demyelinating polyneuropathy (CIDP) in addition to emphysema. In addition, patient 76430 presented with severe emphysema/COPD and bronchiectasis. Detailed clinical descriptions of these patients will be reported separately. The remaining patients were referred to the DNA_1_ Advanced Alpha-1 Screening™ program by the treating physician due to clinical presentation or symptoms potentially indicative of AATD; i.e., COPD, asthma, emphysema, panniculitis, cerebral aneurysm, or liver disease.

### *SERPINA1* mutations

In this patient cohort, NGS DNA analysis identified 21 separate rare/novel variants. All amino acid changes are reported without the 24 amino acid precursor unless otherwise stated. The following variant types were identified: splice variants (*n* = 2), base pair deletions (*n* = 1), base pair changes resulting in a stop codon (n = 2; one stop codon was found in two patients), and SNVs (*n* = 16; one novel SNV [P28L] was found in two patients) (Table 1).

#### Splice variants

A novel splice variant (G > C) was discovered at position + 1 of intron 1C. The mutation occurred in a patient with no other *SERPINA1* variants but with a low AAT level of 70 mg/dL. A further splice variant (6326c.917 + 1G > A), which resulted in an even lower AAT level of 62.6 mg/dL, was discovered in patient 24023.

#### Base pair deletions

A single base-pair deletion was observed in patient 10724, with a low AAT level of 52 mg/dL. The base pair deletion added 5 heterologous amino acids beyond position 347 before a stop codon, as well as truncating the remainder of the protein.

#### Stop codons

Sequence variants in two siblings (patients 6326 [female] and 6376 [male]), resulted in the insertion of a stop codon at position 156 (stop codon in normal AAT is position 418). Both had additional, well known pathogenic alleles: E342K (Z allele; patient 6326) and R39C (I allele; patient 6376). Combination with the Z allele in patient 6326 resulted in extremely low serum AAT levels (2 mg/dL). A further patient (ID 19771) was found to have a premature stop codon at amino acid 214, which resulted in an AAT level of 91.4 mg/dL.

#### SNVs

Of the 16 rare/novel SNVs found in this investigation, two (found in patients 14271 and 15230) were heterozygous for the known pathogenic Z allele. In addition, two patients (21034 and 24319) were heterozygous for the known pathogenic S allele, and a further patient (23523) was heterozygous for the known pathogenic F allele. One novel SNV occurred twice in siblings (patients 4293 [male] and 5564 [female]) in combination with the known, rare, pathogenic Q_0bellingham_ variant. The remaining novel SNVs (*n* = 10) were heterozygous with the normal M allele or M subtypes (M1, M2, M2/4, etc.), which are secreted in similar concentrations and are comparable in function to the wildtype protein.

### Computational analysis of SNVs

Computational predictions are presented in Table 2. Overall, the agreement between the SVM analysis and the additional computational predictors (FoldX and PolyPhen-2) was strong for all but two SNVs. Exceptions were the Q40R (patient 10889) and H262Y (patient 76430) sequence variants – both were associated with moderate deleterious scores by SVM (0.6589 and 0.6708, respectively), but the sequence variants were not predicted to destabilize the protein (i.e., they had small negative ΔΔG scores that indicate minor stabilization) and were predicted as benign by Polyphen-2.

**Table 2:** Summary of computational analysis of rare/novel SNVs

Patient ID	Novel mutation	Amino acid change	Analysis				
			SVM probability	ΔΔG (FoldX)	PolyPhen-2 Score	Comparison with previous computational characterizations	Comments on side chain structure
Probably deleterious mutations							
1144	P289S^a^	Proline (P; non-polar side chain) > serine (S; polar uncharged side chain)	**0.8282**	**3.49**	**1.000**	• Giacopuzzi et al. (2018) [19] REVEL: **0.901** • VEST3: **0.951** • iFISH: **0.9866** • MutationAssessor: 4.425 (high) • SIFT: **0**	• Tightly packed side chain buried in the same hydrophobic region as M221, in the breach at the top of the A-sheet, and the beginning of the RCL • Serine is not tolerated sterically as it is larger – likely causing disruption to role of this strategic portion of the protein
4668	I50N	Isoleucine (I; hydrophobic side chain) > asparagine (N; polar uncharged side chain)	**0.8153**	**2.69**	**1.000**	Giacopuzzi et al. (2018) [19] • REVEL: **0.873** • VEST3: 0.706 • iFISH: **0.9825** • MutationAssessor: 4.41 (high) • SIFT: **0**	• Highly conserved residue • Polar side chain introduced to a very hydrophobic core of the protein • Will destabilize hydrophobic core
12,642	D341V	Aspartic acid (D; negatively charged side chain) >valine (V; hydrophobic side chain)	**0.8651**	**0.99**	**0.998**	Giacopuzzi et al. (2018) [19] • REVEL: **0.599** • VEST3: 0.765 • iFISH: **0.9823** • MutationAssessor: 4.06 (high) • SIFT: **0.001**	• Conserved residue • Borderline significant change in protein stability • In a buried location found at the “Breach” region of the protein at the base of the RCL loop • Change to valine would eliminate aspartic acid hydrogen bonding to adjacent K343 and possibly affect RCL conformation
14,271	M221 T	Methionine (M; hydrophobic side chain) >threonine (T; polar uncharged side chain)	**0.8186**	**2.93**	**0.997**	Giacopuzzi et al. (2018) [19] • REVEL: **0.933** • VEST3: 0.778 • iFISH: **0.9826** • MutationAssessor: 4.74 (high) • SIFT: **0.001**	• Highly conserved residue • Tightly packed side chain buried in hydrophobic region in the breach at top of α-sheet and beginning of RCL • Threonine would be sterically tolerated due to smaller size but would not have same impact as Methionine on tight packing in strategic area of protein
15,230	V210E	Valine (V; hydrophobic side chain) >glutamic acid (E; negatively charged side chain)	**0.7162**	**1.37**	**0.818**	Giacopuzzi et al. (2018) [19] • REVEL: **0.752** • VEST3: 0.618 • iFISH: **0.9338** • MutationAssessor: 3.745 (high) • SIFT: **0.002**	• Not a highly conserved residue • Residue participates in tight hydrophobic packing near the tip of a β–strand hairpin • Introduction of glutamic acid could cause charge repulsion with D211 and disrupt packing of β-hairpin or could introduce a new h-bond with nearby N390
4293†& 5564†	P28L^a^	Proline (P; non-polar side chain) >leucine (L; hydrophobic side chain)	**0.8205**	**1.17**	**0.648**	Giacopuzzi et al. (2018) [19] • REVEL: 0.387 • VEST3: 0.404 • iFISH: 0.7976 • MutationAssessor: 2.86 (medium) • SIFT: **0.038**	• Highly conserved residue • P28 is near the N-terminus and the side chain packs against P23. • Change to the larger hydrophobic leucine would be sterically permissible as the side chain is surface-accessible. • Possible that the wildtype Proline is necessary to kink the helix for the tight packing to occur, and the conformation of N-terminal helix interaction with the rest of the protein
21,034	P369H	Proline (P; non-polar side chain) >histidine (H; positively charged side chain)	**0.8784**	**3.36**	**1.000**	Giacopuzzi et al. (2018) [19] • REVEL: **0.834** • VEST3: **0.945** • iFISH: **0.9877** • MutationAssessor: 4.755 (high) • SIFT: **0**	• Buried location found at end of the RCL loop • Change to histidine would disrupt packing and affect RCL conformation
24,319	A142D	Alanine (A; small hydrophobic side chain) >aspartic acid (D; negatively charged side chain)	**0.7958**	**1.03**	**0.992**	Giacopuzzi et al. (2018) [19] • REVEL: **0.615** • VEST3: 0.694 • iFISH: **0.9591** • MutationAssessor: 3.51 (high) • SIFT: **0.003** Silva et al., (2016) [20] • PolyPhen-2: **0.99**	• Highly conserved residue • Change to aspartic acid could be sterically problematic as larger charged side chain is introduced to a hydrophobic pocket and could destabilize it
Possibly deleterious mutations							
9533	M385 T	Methionine (M; hydrophobic side chain) >threonine (T; polar uncharged side chain)	**0.8722**	**3.34**	0.134	Giacopuzzi et al. (2018) [19] • REVEL: **0.668** • VEST3: 0.738 • iFISH: **0.801** • MutationAssessor: 1.97 (medium) • SIFT: 0.094	• Conserved residue • Residue in buried core of protein and makes at least 12 hydrophobic contacts in the core • Change to threonine would shorten the side chain and disrupt core packing; note the significant stability drop
21,636	V333 M	Valine (V; hydrophobic side chain) >methionine (M; hydrophobic side chain)	**0.7237**	−0.25	**0.990**	Giacopuzzi et al. (2018) [19] • REVEL: **0.539** • VEST3: 0.676 • iFISH: **0.8378** • MutationAssessor: 1.985 (medium) • SIFT: 0.079 Silva et al., (2016) [20] • PolyPhen-2: 0.53	• Buried location with low ASA; found within the beta-sheet region • Larger/longer side-chain • Methionine may sterically clash in the buried location
Possibly neutral mutations							
2343	I9N [includes precursor]^a^	Isoleucine (I; hydrophobic side chain) > asparagine (N; polar uncharged side chain)	0.3387	N/A	0.517	Giacopuzzi et al. (2018) [19] • REVEL: 0.453 • VEST3: 0.291 • iFISH: 0.3779 • MutationAssessor: 1.1 (low) • SIFT: **0.001**	• Not included in Fig. 1 visualization (no structural information on this portion of the protein)
10,889	Q40R^a^	Glutamine (Q; polar uncharged side chain) >arginine (R; positively charged side chain)	**0.6589**	−0.35	0.018	Giacopuzzi et al. (2018) [19] • REVEL: 0.311 • VEST3: 0.092 • iFISH: 0.5284 • MutationAssessor: 1.515 (low) • SIFT: 0.24	• Conserved residue • Change to the larger Arginine side chain would present steric problems, despite its accessibility • Q40 hydrogen bonds to V302 and while the larger side chain could also hydrogen bond, it could disrupt packing of the helix that holds V302
17,657	K174E^a^	Lysine (K; positively charged side chain) > glutamic acid (E; negatively charged side chain)	**0.5053**	0.21	0.030	Giacopuzzi et al. (2018) [19] • REVEL: **0.622** • VEST3: 0.681 • iFISH: **0.6117** • MutationAssessor: 2.24 (medium) • SIFT: 0.208	• Moderately conserved residue • Change to side chain is sterically tolerated as a smaller side chain is introduced in to a solvent-accessible loop of the protein
76,430	H262Y	Histidine (H; positively charged side chain) >tyrosine (Y; largely hydrophobic side chain, but hydroxyl group can participate in hydrogen bonds or also be phosphorylated)	**0.6708**	−0.68	0.040	Giacopuzzi et al. (2018) [19] • REVEL: 0.086 • VEST3: 0.144 • iFISH: 0.5173 • MutationAssessor: 1.54 (low) • SIFT: **0.042** Silva et al. (2016) [20] • Polyphen-2: 0.06 (21)	• Highly conserved residue • Tightly packed side chain buried • Histidine is involved in 3 hydrogen bonds – to backbone atoms of residues N265, E266, K234 • Tyrosine might not be tolerated sterically because it is larger
Probably neutral mutations							
CA97	E204K^a^	Glutamic acid (E; negatively charged side chain) >lysine (K; positively charged side chain)	0.1021	− 0.70	0.000	Giacopuzzi et al. (2018) [19] • REVEL: 0.457 • VEST3: 0.648 • iFISH: 0.1155 • MutationAssessor: − 0.625 (low) • SIFT: 0.921	• Not a conserved residue, larger lysine chain is sterically tolerated & can make similar contacts. • Little change in protein stability is predicted. Although variant could affect RCL from a distance
23,523	A325P	Alanine (A; small hydrophobic side chain) >proline (P; non-polar side chain)	0.0878	0.72	0.000	Giacopuzzi et al. (2018) [19] • REVEL: 0.214 • VEST3: 0.143 • iFISH: 0.4862 • MutationAssessor: 0.265 (medium) • SIFT: 0.411	• Not conserved • Insignificant change in protein stability • In a surface-accessible loop, that could play a role in an alternate trypsin binding site

All sequence variants are reported without the 24 amino acid precursor unless otherwise stated; ^a^variants previously reported in the dbSNP only

Computational analysis (excludes MutationAssessor): numbers in bold, deleterious score; numbers in normal text, neutral score; *RCL* Reactive center loop, *SVM* Single vector machine, *ASA* Accessible surface area

#### Probably deleterious variants

Eight sequence variants were classified as probably deleterious (i.e., all three predictors registered a deleterious score). Two patients (14271 and 15230) were found to have novel mutations, M221T and V210E, respectively, in combination with the Z allele. Computational analyses strongly suggested that both novel sequence variants were deleterious. The AAT levels found in these samples (47 and 34 mg/dL, respectively) were lower than would be expected for an individual with the PI*MZ genotype [66–100 mg/dL] [8] and were around the range of an individual homozygous for the Z allele [20–45 mg/dL] [1]. Additionally, two siblings (patients 4293 and 5564) presented with low AAT levels and a highly unusual genotype – the known pathogenic rare mutation Q0_bellingham_ was accompanied by the novel mutation P28L, which all three computational analyses predicted to be damaging.

Most of the remaining rare/novel SNVs that the computational analyses predicted to be probably pathogenic were heterozygous with normal alleles. The presence of the P289S (patient 1144), I50N (patient 4668), D341V (patient 12642), or A142D (patient 24319) sequence variants appeared to result in AAT levels ranging from 72 to 89 mg/dL – levels that are often associated with PI*MZ individuals. Moreover, patients 1144 and 4668, who presented with the P289S and I50N mutations, respectively, were recorded as having lung disease.

There was only one exception from the general agreement between computational predictions and AAT serum levels – the P369H mutation, observed in patient 21034. All three computational analyses predicted the mutation to be highly deleterious; however, the AAT serum level was normal (121.2 mg/dL). This may have been due to the presence of an inflammatory state at the time of sampling; unfortunately, a CRP value was not available for this patient.

#### Possibly deleterious variants

Two sequence variants were classified as possibly deleterious (i.e., two of three predictors registered a deleterious score). The variant M385 T (patient 9533) was found in combination with a wildtype allele. The M385 T variant is a good example of how methods that focus on a variety of structural parameters for prediction may be more effective than those that more heavily weight sequence conservation. Both the SVM deleterious result and the FoldX prediction of a significant drop in stability make this variant likely deleterious. These predictions correspond to an AAT level of 73 mg/dL, which was likely to be mainly contributed by the wildtype (normal) allele in this patient. While Polyphen-2 predicted the M385 T variant to be benign, the added structural information considered by both the SVM and FoldX predictors contributed to a greater sensitivity to detect this variant as deleterious. Lastly in this category, the variant V333 M (patient 21636) was found in combination with M3 and M4 alleles and a serum level of 88.4 mg/dL. The SVM and Polyphen-2 predictions were deleterious, while the FoldX score predicted no destabilization of the protein.

#### Possibly neutral variants

Four sequence variants were predicted to be possibly neutral (only one of the three predictors scored as deleterious). The I9N (includes precursor) found in patient 2343 was classified as possibly neutral as the SVM prediction (0.3387) was below the deleterious threshold, accompanied by a borderline pathogenic score (0.517) from PolyPhen-2, and a moderate AAT level of 86 mg/dL. As this mutation is in the cleaved precursor region of AAT and as there are no coordinates for this residue in the protein structure, a Gibbs free energy change cannot be calculated. The Q40R variant had an SVM score of 0.6589 (a moderately deleterious result), but was not predicted to destabilize the protein, and scored benign by Polyphen-2. This variant was accompanied by a second M3 allele, and serum AAT levels were not obtained for the patient (10889). A novel mutation from patient 17657 (K174E) was predicted by SVM to have a borderline deleterious score of 0.5053; however, the score had ±0.036 standard deviation and could thus potentially be below the threshold for deleterious. This was accompanied by benign predictions by FoldX and Polyphen-2, and was associated with normal AAT levels (160 mg/dL). The H262Y variant (patient 76430) was associated with a moderate deleterious prediction by SVM (0.6708), but was not predicted to destabilize the protein, and was predicted benign by Polyphen-2. Nonetheless, the low serum AAT level of 74.8 mg/dL found in this patient accompanied by the presence of lung disease are suggestive of deleterious effects.

#### Probably neutral variants

In this last category, two variants were predicted to be probably neutral (i.e., none of the three predictors scored as deleterious). One mutation in a non-conserved residue (E204K) found in patient CA97 was predicted to be neutral by all predictors – this is supported by the normal AAT level found in this patient (112 mg/dL). Similarly, the A325P mutation (patient 23523) was accompanied by a normal AAT level of 118.6 mg/dL, with agreement among the three predictions that the mutation was neutral.

### Benchmarking of SVM predictions

Table 3 provides the SVM predictions for each of the benign and pathogenic variants included in the benchmarking analysis, with comparisons to PolyPhen2 and FoldX predictions. For the pathogenic variant set, 17/17 (100%) were predicted to be deleterious by SVM, and correspondingly 16/17 variants (94.1%) were predicted to have negative effects on stability of the protein by FoldX. PolyPhen2 predicted 16/17 (94.1%) to be pathogenic. For the benign ClinVar set, 5/5 variants (100%) were predicted by both the SVM and PolyPhen-2 to be benign, and correspondingly 4/5 were predicted by FoldX to slightly improve protein stability (negative values indicate better predicted stability with the variant). In the alternative benign primate dataset, 28/35 variants (80%) were predicted to be benign by the SVM, compared with 32/35 predicted (91.4%) predicted to be benign by PolyPhen-2. The statistical parameters calculated suggest that the accuracy of both SVM and PolyPhen-2 predictors are broadly similar (Table 4). While the sample sizes of this benchmark set are not sufficient for a comprehensive comparison of the SVM to Polyphen-2 or other predictors, overall the benchmark testing on these variants of known effect on *SERPINA1* function validates the strength and accuracy of the SVM and Polyphen-2 for predictions on novel variants presented in this work.

**Table 3:** Results of benchmarking analysis

*SERPINA1* mutation	Species	Sequence Identity (%)	SVM probability	ΔΔG (FoldX)	PolyPhen-2 Score
Pathogenic sequence variants (ClinVar)					
P369L	*Homo sapiens*	100	**0.8496**	**1.510**	**1.0000**
P369S	Homo sapiens	100	**0.8353**	**4.410**	**1.0000**
P369T	Homo sapiens	100	**0.8599**	**2.810**	**1.0000**
M358R	Homo sapiens	100	**0.7853**	0.431	0.0190
E342K	Homo sapiens	100	**0.8413**	**2.090**	**1.0000**
E264V	Homo sapiens	100	**0.8619**	**1.660**	**1.0000**
D256V	Homo sapiens	100	**0.8708**	**1.950**	**0.9850**
G225R	Homo sapiens	100	**0.8975**	**5.160**	**0.9820**
R223C	Homo sapiens	100	**0.8954**	−0.350	**0.9950**
I92N	Homo sapiens	100	**0.8215**	**3.600**	**1.0000**
G67E	Homo sapiens	100	**0.8680**	**26.290**	**1.0000**
S53F	Homo sapiens	100	**0.8687**	**19.860**	**1.0000**
L41P	Homo sapiens	100	**0.7585**	**3.240**	**0.6010**
R39C	Homo sapiens	100	**0.8672**	**2.110**	**1.0000**
A336T	Homo sapiens	100	**0.8479**	**3.450**	**1.0000**
G115S	Homo sapiens	100	**0.7826**	**1.610**	**0.9990**
F52S	Homo sapiens	100	**0.7900**	**6.020**	**1.0000**
Benign sequence variants (ClinVar)					
E376D	Homo sapiens	N/A	0.2991	**1.850**	0
E363K	Homo sapiens	N/A	0.4172	−0.900	0.11
A284S	Homo sapiens	N/A	0.3445	−0.240	0.139
V213A	Homo sapiens	N/A	0.1161	−0.100	0
R101H	Homo sapiens	N/A	0.0576	−0.533	0
Benign sequence variants (Primate neutral variants)					
P21Q	Hylobates sp. ECACC	95	*0.363*	*0.72*	0.014
F23L	Papio anubis	92	0.3762	0.38	0
T27A	*Gorilla gorilla*	98	0.4118	**1.34**	0
N29K	Hylobates sp. ECACC	95	0.4365	−0.63	0.178
N29S	Papio anubis	92	0.0839	0.13	0
T48S	Hylobates sp. ECACC	95	0.4991	0.51	0
I50V	Gorilla gorilla	98	**0.6687**	*0.96*	**0.767**
D74S	Chlorocebus sabaeus	92	0.0588	0.38	0
N81H	Pongo abelii	96	0.462	0.27	0.007
I92V	Pongo abelii; Hylobates sp. ECACC	96; 95	**0.7931**	*1.06*	0.006
Q105K	Papio anubis	92	0.2178	−0.5	0.001
N116S	Gorilla gorilla	98	**0.751**	**1.9**	0.311
K136N	Papio anubis	92	0.4292	0.07	0
E141D	Pongo abelii	96	0.4052	0.77	0.002
G148E	Chlorocebus sabaeus	92	0.219	−0.46	0
D159N	Papio anubis	92	0.165	−1.21	0
Q212E	Pongo abelii	96	0.2568	0.35	0
V213A	Hylobates sp. ECACC	95	0.1161	−0.1	0
Q230Y	Papio anubis	92	0.0803	0.49	0
Q230H	Hylobates sp. ECACC	95	0.0545	0.41	0
K233E	Papio anubis	92	0.0787	0.58	0
D270E	Hylobates sp. ECACC	95	0.2543	−0.03	0
I271V	Gorilla gorilla	98	0.1015	0.52	0
D280N	Pongo abelii	96	0.1407	*0.82*	0
S285N	Chlorocebus sabaeus	92	0.0554	−1.6	0
S292A	Chlorocebus sabaeus	92	0.3206	−0.14	0.003
S301R	Pongo abelii	96	0.1328	−0.54	0.011
S301T	Hylobates sp. ECACC	95	0.0724	0	0
S313G	Hylobates sp. ECACC	95	**0.606**	*0.63*	0.05
E324D	Chlorocebus sabaeus	92	0.0978	*0.62*	0
A332V	Homo sapiens	99	0.1255	**1.88**	**0.99**
I360V	Gorilla gorilla	98	0.1547	*0.73*	0
L383H	Hylobates sp. ECACC	95	**0.8834**	**3.08**	**1**
M385I	Papio anubis	92	**0.7686**	**3.15**	0.001
M385 V	*Pan troglodytes*; Pongo abelii	99; 96	**0.7566**	**2.44**	0.001

All variants are reported without the 24 amino acid precursor unless otherwise stated

Scores in bold, pathogenic; scores in italics, possibly pathogenic; scores in normal text, benign. *SVM* Support vector machine

**Table 4:** Measurements of benchmarking predictions

	TPR (sensitivity)	TNR (specificity)	PPV	NPV	ACC	BACC
SVM	1.0	0.825	0.720	1.0	0.879	0.913
Polyphen2	0.944	0.925	0.85	0.974	0.931	0.935

*TNR* True negative rate, *TPR* True positive rate, *PPV* Positive predictive value, *NPV* Negative predictive value, *ACC* Total accuracy, *BACC* Balanced accuracy

## Discussion

Through the DNA_1_ Advanced Alpha-1 Screening™ program, we have begun to encounter large numbers of novel sequence variants of the *SERPINA1* gene, as evidenced by the data we have presented. The present study is supportive of several earlier investigations that have uncovered previously uncharacterized and potentially pathogenic sequence variants of *SERPINA1* [7, 9, 12, 19, 21]. There is a growing body of evidence to suggest that novel sequence variants may be more clinically impactful than previously thought, with some reported to be associated with early onset COPD [9].

Using NGS, we identified 21 rare/novel sequence variants of the *SERPINA1* gene in patients suspected of having AATD. Most of the variants (*n* = 16) were SNVs. In addition, two base pair changes resulting in stop codon insertions, one base pair deletion, and two splice variants were discovered. All of the SNVs were previously recorded in the National Center for Biotechnology Information’s database of single nucleotide polymorphisms (dbSNP) and/or in the literature [19, 20, 22–26] (Table 1). The I50N variant (Pi_Tijarafe_) was previously confirmed as pathogenic in an vitro cell model, and was associated with similar AAT expression to the Z variant [26]. Nonetheless, to the best of our knowledge, this is the first study to describe seven variants (E204K, P289S, Q40R, M221T, K174E, I9N [includes precursor] and P28L) alongside additional patient data. However, despite the availability of other data such as AAT levels, determining whether these variants are clinically relevant is challenging. We therefore sought to evaluate the utility of computational modeling to provide supporting evidence, in addition to observed AAT serum levels, of the pathogenicity of rare SNVs. We note that computational methods predict the effects of missense variants on either protein function (SVM, and machine learning approaches) or the inherent stability of the tertiary/quaternary structure of a protein (FoldX). However, this may not always correspond with clinical parameters, such as secreted protein serum levels, or the degree of pathogenicity in a particular organ.

**Fig. 3:**
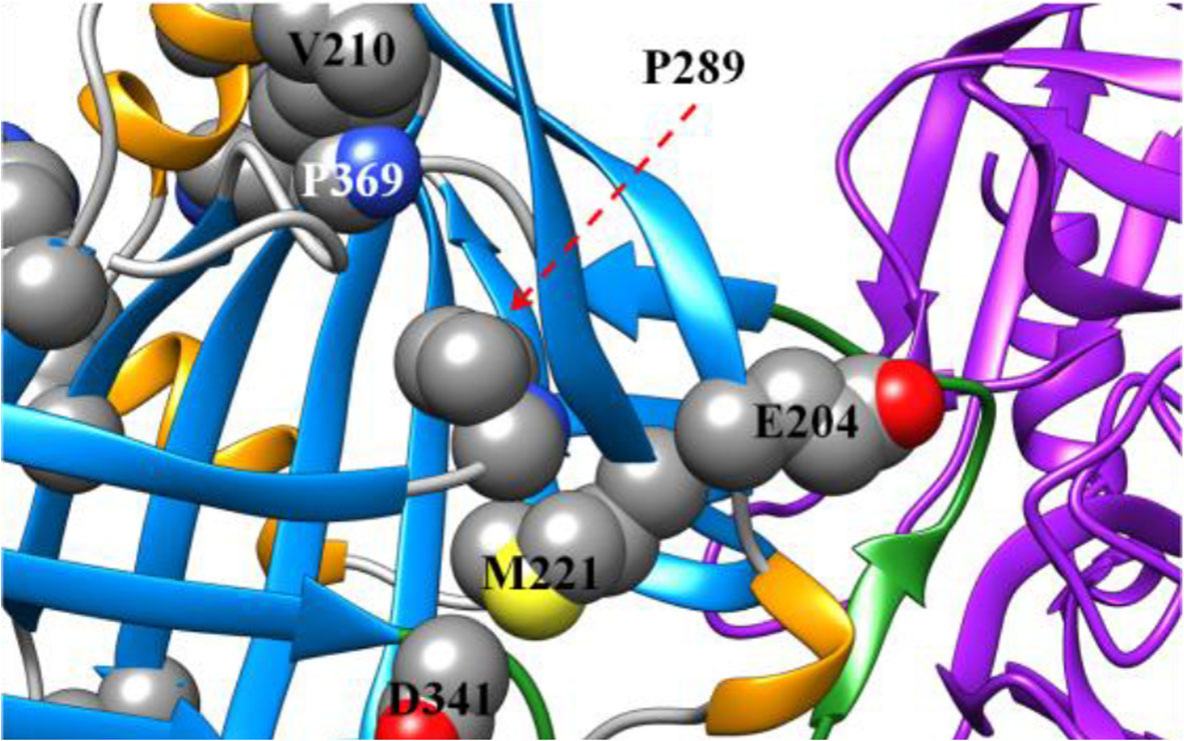
Structure of AAT zoomed in on locations of interest. Presented are some of the missense residues predicted to negatively affect the stability of the folded protein. Several of these missense changes are in the tightly packed core of the protein, such as the P289S variant packed tightly near the M221T variant location

The majority of the sequence variants identified in our cohort were predicted to be deleterious by computational methods. Only two variants were predicted to be probably neutral by all three computational techniques. Of the rare variants previously reported in the dbSNP only (E204K, P289S, Q40R, M221T, K174E, I9N [includes precursor] and P28L), the probably deleterious variants were predicted to be, P289S, M221T, and P28L, and were accompanied by low AAT levels. In particular, the P289S variant was found in a 61-year-old patient with advanced emphysema, supporting the pathogenicity of this variant. The remaining variants were predicted to be neutral or possibly neutral, and were accompanied by normal or low-normal AAT levels (although no AAT level was reported with the Q40R variant), and are less likely be clinically relevant. Although there is some evidence of a relationship between AAT variants and cerebral aneurisms [27], we do not have sufficient evidence to conclude a causal relationship between the clinical presentation in patient (CA97) and the E204K variant. For the rare variants predicted to be probably deleterious or possibly deleterious, in line with previous reports, we observed that the majority of these cluster around functional domains of AAT [20]. The mechanism of pathogenicity for most of these sequence variants (I50N, P289S, M385T, M221T, D341V, V210E, P369H, V333M and A142D) is likely to be via disruption of the tightly packed hydrophobic core of the AAT protein, and some may in turn disrupt the adjacent reactive center loop (RCL; Fig. 3) that inhibits proteases. One possible mechanism is that substantial changes to the core of the protein could result in misfolding of the protein within hepatocytes, such that only small amounts of AAT would be released, resulting in reduced levels of AAT in the peripheral circulation. An alternative mechanism of pathogenicity might include missense changes that do not affect AAT folding and result in normal levels detected in serum, but have a deleterious effect on conformational changes required for sheet opening or protein-protein interactions necessary for inhibition of neutrophil elastase.

As expected, very low blood levels of AAT were found in heterozygotes for known deficiency alleles and new mutations. Two patients (12230 and 15230) in this study had very low AAT levels around the range associated with a PI*ZZ individual [20–45 mg/dL] [1], and novel pathogenic variants in combination with the Z allele. Patients such as these would be strong candidates for AAT therapy if they presented with airflow obstruction and significant emphysema [28]. There are more than 6 million individuals in the United States alone with the PI*MZ genotype [5]. As shown by this study, it is possible that numerous other patients may be undiagnosed compound heterozygotes with rare/novel sequence variants not detectable by IEF or targeted genotyping. The concept of cumulative deleterious effects in compound heterozygotes has previously been described for the PI*FZ genotype [29]. The F allele is associated with normal AAT levels but reduced AAT functionality, while low circulating levels are observed in Z patients [29]. All AAT secreted by PI*FF homozygotes has reduced functionality and these individuals have been shown to be at increased risk of lung damage caused by uninhibited elastase [29]. In PI*FZ heterozygotes, functionality and circulating levels are both reduced, resulting in an increased risk of emphysema compared with PI*FF patients [29].

Most novel sequence variants within our cohort were heterozygous with normal variants; it is therefore difficult to fully assess the impact of these variants on serum AAT levels and risk of emphysema. For known variants the disease risk is well known. For example, individuals with the PI*MZ genotype have a greater degree of airflow obstruction than PI*MM individuals with comparable smoke exposure, and ever-smoking PI*MZ individuals have an increased risk of developing COPD [30]. However, the longitudinal disease-risk associated with rare alleles is unknown and AAT levels, although indicative of severity, are not conclusive. As the majority of these rare/novel variants will probably have different mechanisms of pathogenicity, it is possible that the disease risk is different to that of common heterozygotes and is specific for each variant. Further biochemical and clinical characterization is needed to fully understand how these sequence variants contribute to lung disease.

AATD is usually associated with single amino acid substitutions/deletions leading to subtle structural changes to the AAT protein; however, this study also identified splice variants, stop codons, and large deletions in *SERPINA1*. The potential contribution of these sequence variants to AATD should not be underestimated, especially when occurring in combination with damaging structural mutations. For example, in patient 6326, insertion of a stop codon at position 156 in combination with the Z mutation resulted in a severe reduction in antigenic AAT levels (2 mg/dL). This effect was not apparent in this patient’s sibling (patient 6376), whose AAT level was 98 mg/dL. Patient 6376 is heterozygous for the above mentioned stop codon and the PI*I (R39C) allele – the PI*I mutation gives rise to a misfolded AAT protein, which is present in peripheral blood at near-normal concentrations [31]. This further demonstrates that rare and novel sequence variants can become more clinically relevant in combination with common deficiency alleles.

For patients with rare/novel mutations, apart from instances where the variants are deletions or null variants, it can be difficult to determine the impact of sequence variants and if treatment with exogenous AAT is necessary. This study has demonstrated that computational analyses may be useful in understanding the potential impact of novel mutations. The three predictive computational methods presented were generally in agreement and in most cases related to the observed AAT levels. In particular, we found that the enhanced structural information that contributes to the SVM predictions may confer a greater sensitivity to deleterious variants, making it suited for clinical genetics applications. The benchmarking analysis provides a strong validation for the balanced accuracy of the SVM predictions and supports its use in predicting the effects of the novel variants described in the current work. In addition, there was good agreement between results of the present analysis and previous studies [19, 20] (Table 2). One exception to the general agreement between this and previous studies may be P28L, with other computational measures suggesting that it is of intermediate pathogenicity. However, it is notable that the number of previously reported deleterious scores generally mirror that of those reported in the present study through the categories of probably deleterious, possibly deleterious, possibly neutral and probably neutral utilized in the present study. In particular, in the probably neutral section, no deleterious scores are presented from this analysis or previous reports.

Some important limitations of this study should be mentioned. This observational study was not controlled, i.e., there were no formal inclusion and exclusion criteria and no control group, and data were collected from a small (*N* = 23) patient population. In addition, genetic and non-genetic factors – not related to the AAT sequence variants reported here – may have contributed to the development of COPD. However, these factors are beyond the scope of the current report. Furthermore, computational modeling of missense variants only predicts if a substitution is deleterious to protein function or stability. We do not know the exact mechanisms by which these substitutions lead to either reduced AAT levels or weakened elastase-inhibiting activity. Furthermore, it should be noted that a host of different modeling software are available, and each may produce different results for a particular mutation, as demonstrated by Giacopuzzi et al. (Table 2). It was outside the scope of the present study to assess a wide range of modeling techniques, as a further aim of the study was to relate the computational scores to clinical parameters. However, Giacopuzzi et al. raise an important point, in that no individual computational method is infallible, and in an ideal situation, more than one technique should be consulted in the clinical decision-making process. In addition, computational predictions may be inconsistent with findings of experimental characterization; therefore, ultimately, detailed biochemical functional analysis of the protein is required to validate the findings of computational analyses. In addition, clinical information on patient presentation is required in order to obtain a full picture of the patient’s individual disease risk.

Despite the above limitations, this study demonstrates that there are numerous potentially pathogenic novel variants beyond those commonly associated with AATD. Due to the progressive and irreversible destruction of lung tissue seen in severe AATD, early and accurate diagnosis is crucial to prevent further loss of lung tissue. Data from the RAPID/RAPID Extension trials has demonstrated that while treatment with AAT can slow the loss of lung tissue, tissue lost prior to commencing treatment cannot be regained [32, 33]. This is compounded by the fact that patients often experience long delays before receiving an accurate diagnosis [34], partly due to a lack of specialized testing. Early diagnosis also enables patients to implement lifestyle changes such as smoking cessation and avoidance of passive smoke. However, identifying rare/novel variants can be difficult, and this task may be impossible by traditional methods such as protein phenotyping via IEF [10].

The increasing availability of commercial DNA testing is helping to improve diagnosis of patients with AATD and rare genotypes [35]. However, many current approaches do not incorporate sequencing, and are unable to detect potentially pathogenic rare/novel variants that may lead to development of AATD. The need for faster screening and diagnosis of AATD has led to the development of the DNA_1_ Advanced Alpha-1 Screening™ Program. DNA_1_ testing incorporates AAT levels, C-reactive protein serum levels, targeted genotyping (including the F and I alleles), and IEF, and reflexes to NGS when these methods prove insufficient. Our results support the proposal by Graham et al*,* who recommended that individuals with low serum levels and no resolution in targeted tests should be subjected to full-gene sequencing [12].

## Conclusions

Advancements in DNA sequencing technology continue to reveal numerous rare/novel sequence variants in the *SERPINA1* gene. Many of these variants may be pathogenic and causative factors in the development of AATD. Computational modeling opens new dimensions of structural analysis, which can help to define the pathogenic nature of these variants more accurately. The computational analyses we present are straightforward to perform and can provide a valuable additional indication (in combination with serum levels and clinical presentation) of the pathogenicity of novel mutations. We expect that this added information will eventually lead to improved individualized therapy for patients with AATD.

## Additional file

Additional file 1: Protein modeling to assess the pathogenicity of rare variants of SERPINA1 in patients suspected of having Alpha 1 Antitrypsin Deficiency (DOCX 25 kb)

## Data Availability

All sequencing data reported have been deposited within a publicly accessible database (NCBI BioProject; Accession: PRJNA547351; URL: https://www.ncbi.nlm.nih.gov/bioproject/?term=PRJNA547351). All other available data are reported within this manuscript and its Additional file.

## Abbreviations

AAT: Alpha 1 Antitrypsin
AATD: Alpha 1 Antitrypsin Deficiency
IEF: Isoelectric focusing
NE: Neutrophil elastase
NGS: Next-generation sequencing
RCL: Reactive Center Loop
SNP: Single-nucleotide polymorphism
SNV: Single-nucleotide variation
SVM: Support vector machine
