# Supplementary material for: Protein modeling to assess the pathogenicity of rare variants of SERPINA1 in patients suspected of having Alpha 1 Antitrypsin Deficiency
Source: BMC Med Genet. 2019 Jul 15;20:125. doi: 10.1186/s12881-019-0852-5 (PMC6631921; doi:10.1186/s12881-019-0852-5)
Supplement: Supplementary file 1 — Protein modeling to assess the pathogenicity of rare variants of SERPINA1 in patients suspected of having Alpha 1 Antitrypsin Deficiency (DOCX 25 kb) [file 12881_2019_852_MOESM1_ESM.docx]

**Protein modeling to assess the pathogenicity of rare variants of *SERPINA1* in patients suspected of having Alpha 1 Antitrypsin Deficiency**

Friedrich Kueppers, Mark D. Andrake, Qifang Xu, Roland L. Dunbrack, Jr., Joannah Kim, Christopher L. Sanders

**Features of Support Vector Machine (SVM) classifier in mutation prediction**

*Position-specific scoring matrix (****PSSM****)*

The PSSM captures the conservation pattern in alignment and stores it as a matrix of scores for each position. Highly conserved positions have high scores and weakly conserved positions have scores near zero. PSSMs were calculated from the multiple sequence alignments from PSI-BLAST (1) searches of uniref100 database – PSSM values were taken from the second round of search with E-value cutoff set at 10. The difference between the PSSM score of wildtype residue and the PSSM scores of mutant residues is termed dPSSM. Larger dPSSM scores mean that the mutant deviates more from the wildtype and more likely to be deleterious; a smaller dPSSM suggests that the mutant is similar to wildtype.

*Conservation score (****Con_Score****)*

BLASTCLUST was used to cluster the PSIBLAST output using a sequence identity threshold of 35% (2) –conservation score was calculated from AL2CO (3). The sequences in the cluster containing the query were created with the MUSCLE program (4). The multiple sequence alignment was input in to the AL2CO program to calculate the conservation score.

*Accessible surface area of the mutation site in a biological assembly (****SA_BA****)*

Calculated by the NACCESS program (5). If the amino acid could be presented in the coordinates of more than one associated structure, then the minimal surface was used as the surface area of that mutation. A smaller surface area implies the residue is likely in the interface or core of the protein, this a mutation is more likely to be deleterious.

*Accessible surface area (ASA) of the mutation site in a monomer (****SA_Monomer****)*

From coordinate files containing only a single protein with no biological assembly partners or ligands. A smaller surface area means the residue is likely in the core of the protein, so a mutation is more likely to be deleterious.

*Other features include:*

- *Side chain volume change between wildtype and mutant* (***SC_volume***)
- *Hydrophobic change between wildtype and mutant* (***HPhobic***)
- *The disorder probability from VSL2 program (****VSL2_dis****) –* higher probability value, more likely disordered (6)
- *Disorder probability from IUpred program* *(****IUpred_dis****)* – higher probability value, more likely disordered (7)
- *Disorder probability from Espritz program (****Espritz_dis****)* – higher probability, more likely disordered (8)

**References**

1. Wei Q, Xu Q, Dunbrack RL, Jr. Prediction of phenotypes of missense mutations in human proteins from biological assemblies. Proteins. 2013;81:199-213.

2. Altschul SF, Madden TL, Schaffer AA, Zhang J, Zhang Z, Miller W, et al. Gapped BLAST and PSI-BLAST: a new generation of protein database search programs. Nucleic Acids Res. 1997;25:3389-402.

3. Pei J, Grishin NV. AL2CO: calculation of positional conservation in a protein sequence alignment. Bioinformatics. 2001;17:700-12.

4. Edgar RC. MUSCLE: multiple sequence alignment with high accuracy and high throughput. Nucleic Acids Res. 2004;32:1792-7.

5. Hubbard SJ and Thornton JM. ‘NACCESS’, computer program. 1993. Department of Biochemistry and Molecular Biology, University College, London.

6. Vucetic S, Brown CJ, Dunker AK, Obradovic Z. Flavors of protein disorder. Proteins. 2003;52:573-84.

7. Dosztanyi Z, Csizmok V, Tompa P, Simon I. The pairwise energy content estimated from amino acid composition discriminates between folded and intrinsically unstructured proteins. J Mol Biol. 2005;347:827-39.

8. Walsh I, Martin AJ, Di Domenico T, Tosatto SC. ESpritz: accurate and fast prediction of protein disorder. Bioinformatics. 2012;28:503-9.
